# A Systematic Evaluation of *Angelica sinensis* Discrimination Based on FT-MIR Spectroscopic Analysis Combined with Machine Learning

**DOI:** 10.3390/foods15091606

**Published:** 2026-05-06

**Authors:** Lipeng Zhou, Fang Ma, Yifan Yan, Jiulong Yan, Zilong Zhao, Zhirong Sun

**Affiliations:** 1 School of Chinese Materia Medica, Beijing University of Chinese Medicine, Beijing 102488, China; 20240941476@bucm.edu.cn (L.Z.); 202253008@bucm.edu.cn (F.M.); 20240935275@bucm.edu.cn (Y.Y.); bucm19357489290@163.com (J.Y.); 2 Gansu Longcuiyuan Herbal Medicine Co., Ltd., Dingxi 748400, China; 13209327410@163.com

**Keywords:** *Angelica sinensis*, middle-infrared spectroscopy, geographic origin, machine learning, qualitative models

## Abstract

*Angelica sinensis* (Oliv.) Diels (AS) is a medicinal and food plant that has long faced a persistent challenge: its quality and price are often influenced by environmental conditions and geographical origins. To achieve substantial profits, items that are not produced in primary regions, along with counterfeit products, are frequently misbranded as originating from main production areas; this leads to fraud regarding geographic origin and product tampering. Rapid, effective and feasible methods for distinguishing the geographic origin of AS are important for ensuring consumer safety and protecting their interests. This study establishes the authenticity and geographical origins of AS. Meanwhile, diverse machine learning strategies are used to identify the optimal combination by incorporating spectral pre-processing techniques, feature wavenumber selection methods and classification algorithms. The findings reveal that the backpropagation neural network (BPNN), convolutional neural network (CNN) and radial basis function neural network (RBF) excel in determining the authenticity of AS. To distinguish among different growing environments of AS, three models obtained 98.94% classification accuracy on the test set: (1) multiplicative scatter correction (MSC) pre-processing with an RBF classifier, (2) standard normalised variate (SNV) pre-processing with an RBF classifier and (3) Savitzky–Golay (SG) smoothing pre-processing, competitive adaptive reweighted sampling (CARS) for selecting features and a BPNN for classification. This study validates the feasibility of ensemble learning combined with MIR for discriminating AS from authenticity and different geographical sources.

## 1. Introduction

*Angelica sinensis* (Oliv.) Diels (AS), a widely used medicinal and food resource, grows in cold, moist hillside regions at altitudes ranging from 2200 to 3000 m [1]. The dried root of AS (commonly referred to as Danggui in Chinese) has been historically utilised for its capacity to replenish blood, enhance blood circulation, regulate menstrual flow, alleviate pain and facilitate bowel movements, particularly for treating gynaecological conditions [2], owing to its outstanding medicinal value in traditional Chinese medicine theory. In contemporary medical studies, AS has been reported to be used for addressing anaemias [3] and cardiovascular disorders [4], alleviate pain and decrease inflammation [5,6], support the management of fatty liver [7] and boost immune function [8], as it is rich in bioactive substances, such as polysaccharides [9], essential oils, coumarins [10], organic acids [11], and flavonoids [9] ultimately leading to beneficial health outcomes.

AS has a history of ~1700 years of artificial cultivation in China. Owing to increased commercial output and export requirements, the area utilised for AS cultivation now exceeds 43,500 ha [12]. AS in Min County, Dingxi City, and adjacent regions, commonly known as Mingui, is a geographical indication product of Min County, Gansu Province, China. Concurrently, Min County, located in Gansu Province, is the leading area for AS manufacturing in China, accounting for 70% of the overall production [13]. The introduction and cultivation of AS in the provinces of Qinghai, Yunnan and Sichuan have reached a considerable scale. Regrettably, increasing costs associated with AS cultivation and market demands have led to widespread indiscriminate planting. A series of issues are gradually becoming apparent, such as the increasing incidence of bolting, a decline in provenance quality, deviations from standard planting practices and aggressive mining strategies aimed at capturing market share. Such factors reduce AS content or subpar medicinal components [12]. Meanwhile, in pursuit of marked profits, products from regions outside the primary production areas [14] as well as counterfeit goods that are often falsely labelled as Mingui and claimed to originate from the primary production zones, contribute to geographic origin deception and product adulteration [15]. Therefore, effective and reliable quality traceability of AS is crucial for safeguarding the authenticity of Chinese herbal medicine brands, thereby facilitating market globalisation, ensuring the safety assessment of food and medicine and protecting consumers’ essential rights.

Recently, middle-infrared spectroscopy (MIR) has been considered a fast, nondestructive, convenient and accurate detection method for the geographical authentication of food, agriculture and medicine [16]. For instance, Han et al. [17] combined FT-MIR with machine learning to explore the feasibility of ensemble learning for discriminating wild *Paris polyphylla* var. Yunnanensis. Their study demonstrates that the MIR technique is a powerful, fast and effective tool for determining geographical origin. In most cases, a comprehensive assessment of the source and quality of AS cannot be performed via analysing the composition of a single chemical owing to its inherent complexity, which necessitates the consideration of multiple ingredients and indicators. However, the application of MIR data in conjunction with machine learning facilitates the identification of origins and enables the reliable characterisation of plant components and geographical variations [18].

Nevertheless, no existing studies have integrated FT-MIR spectroscopic analysis with machine learning for the identification of AS species and their origins. Furthermore, our previous study indicates that although AS has demonstrated promising predictive capabilities in chemometric classification analysis for different bolting times during the medicine formation period using ATR-FTIR [19], there remains a considerable scope for improvement in systematic investigations of optimisation procedures. This limitation persists as a common issue in predictive models of various herbal and food varieties as well as their origins. Two notable challenges exist in the integrated models that combine FT-MIR spectroscopic analysis with machine learning. First, an integrated, system-wide optimisation strategy necessitates a comprehensive system analysis that encompasses spectral pre-processing techniques, characteristic wavenumber selection methodologies and classification processes modelling [20,21]. A systematic evaluation of AS species discrimination and origin identification shall focus on the inherent synergies between these interdependent techniques. However, the abovementioned methods exhibit distinct evaluation frameworks; studies comparing these methods are scarce in systematic optimisation. Thus, the feasibility of ensemble learning with FT-MIR can be validated by focusing on improving the accuracy, precision, recall and F1 score for each class, thereby enhancing authentication and fraud prevention for AS [22]. Such endeavours shall establish a methodological foundation for accurate geographical traceability.

To address the aforementioned challenges, this study develops a structured optimisation method to effectively ascertain authenticity and geographical origin using FT-MIR and machine learning. A comprehensive spectroscopy dataset comprising counterfeit and authentic AS samples collected from five distinct geographical regions was constructed. The effects of spectral pre-processing techniques (including raw spectra), characteristic wavenumber selection across all three methodological categories and classification algorithms (including parameter optimisation methods) are compared and examined, particularly with respect to the model’s performance and predictive capabilities. This strategy offers a promising approach to species verification and to improving the AS quality assessment framework.

## 2. Materials and Methods

### 2.1. Sample Collection and Preparation

314 batches of AS samples analysed in this study were collected from the following five key production areas in China: Min County (GSMX), various locations in Gansu Province (GSQT), Qinghai Province (QH), Yunnan Province (YN) and Sichuan Province (SC). Thereafter, 70 fake product (FA) samples were collected from different tea plant species, such as *Heracleum candicans* Wall. ex DC, *Angelica gigas* Nakai, *Levisticum officinale* W. D. J. Koch, *Angelica acutiloba* (Sieb. et Zucc.) Kitagawa, *Angelica tsinlingensis* K. T. Fu, *Angelica pubescens* Maxim. f. *biserrata* Shan et Yuan and *Vicatia thibetica* H. Boissieu. The roots of these samples were washed, cut into pieces and oven-dried at 60 °C. All the dried roots were processed into homogeneous powders using a No. 4 sieve with a screen size of 0.25 mm.

### 2.2. MIR Spectra Acquisition

Desiccated roots were ground to a uniform powder by passing them through a No. 4 sieve exhibiting a mesh size of 0.25 mm. KBr crystals of 99% purity were obtained from Zigong Chemical Research and Design Institute (Sichuan, China). The powdered sample was carefully blended with potassium bromide (KBr) and subsequently pressed into a round disc no thicker than 1 mm. The standard MIR spectrum and differential thermal gravimetric analysis (DGTA) data were acquired using a spectral transmission attachment, with a pure KBr film being the background reference. The spectrum was obtained by co-adding 16 scans in the range of 4000–400 cm^−1^ at a resolution of 4 cm^−1^. This resolution is standard for condensed-phase solid samples, as higher resolutions provide negligible improvement due to intrinsic peak broadening [23] while compromising signal-to-noise ratio the number of scans was chosen to achieve a sufficient signal-to-noise ratio within a practical acquisition time [24]. Automatic corrections were applied to the data to determine the effects of water vapour and carbon dioxide. Spectrum v10.5 software (PerkinElmer, Waltham, MA, USA) was used for spectral analysis. The original MIR spectral data were converted to absorbance values and automatically corrected for baseline.

### 2.3. Sample Set Division and Spectral Pretreatment

In the identification of the authenticity of AS, the 314 AS and 70 FA samples obtained were partitioned into training and prediction sets following a 7:3 ratio for subsequent model development and evaluation. Similarly, to identify geographical origins of 314 AS samples, a 7:3 ratio was used to partition into training and prediction sets. Thus, the Kennard–Stone (KS) algorithm was employed to divide the previously mentioned dataset. The KS algorithm operates on the principle that all samples are initially considered in training group, with samples being methodically selected for inclusion in this group. Initially, the pair of samples with the largest Euclidean distance was selected to be added to the training set [25]. Subsequently, the Euclidean distance between each remaining sample and every known sample in the training set was measured. On the basis of these calculations, the samples that were farthest and nearest to the selected samples were identified and incorporated into the training set. The process continued until the desired number of samples was obtained. To further assess the robustness of the KS-based split, 50 independent random stratified splits (70/30, preserving origin proportions) were additionally performed, and the optimal modeling pipeline was reapplied to each split. The Euclidean distance was calculated as follows:

(1) $$d_{x}\left(p,q\right)=\sqrt{\sum_{j=1}^{N}{\left[x_{p}\left(j\right)-x_{q}\left(j\right)\right]}^{2}};\;p,q\in \left[1,N\right],$$

where *X_p_* and *X_q_* denote two distinct samples and *N* signifies the count of spectral points within the sample.

Spectral data acquisition could be affected by multiple factors, such as environment, light scattering, interference and operator variability. As experimental errors are unavoidable in spectral acquisition, appropriate spectral pretreatment is often effective in comprehensively improving the model’s performance before model development. To enhance the model’s stability and improve its ability to generalise, different pre-processing techniques were employed, such as Savitzky–Golay (SG) smoothing [26], multiplicative scatter correction (MSC), standard normal variate (SNV), first derivative (D1), second derivative (D2), Savitzky–Golay first derivative (SG-FD) and Savitzky–Golay second derivative (SG-SD) [27]. Subsequently, different classification algorithms would automatically select the optimal pre-processing method [28].

### 2.4. Characteristic Wavenumber Selection and Parameter Optimisation

The integration of spectral pre-processing techniques and classifiers posed challenges during model development owing to the considerable time and resources required for handling huge amounts of spectral data. A large volume of data could contain redundant or irrelevant information and overlapping spectral data, thereby increasing the model’s complexity. To address such issues, specific information selection techniques [29] had been developed to improve model effectiveness. The techniques aim to enhance computational efficiency and reduce execution time by eliminating unnecessary wavenumbers while retaining the essential ones.

Herein, to streamline the model, boost its precision and reliability and improve its overall effectiveness, six widely used techniques for identifying characteristic wavenumbers were thoroughly applied: competitive adaptive reweighted sampling (CARS), index clear optimise (ICO), random frog (R-Frog), successive projection algorithm (SPA), uninformative variable elimination, uninformative variable elimination (UVE) and variable iterative space shrinkage algorithm (VISSA). CARS dynamically retained the variables that contributed the most to the model through iterative weighted sampling and an adaptive elimination mechanism, notably improving the model’s prediction accuracy and stability [16]. The RF algorithm quantified the selection frequency of each variable through Monte Carlo random sampling and probability evaluation [30]. It identified characteristic wavelengths with high stability and discriminative power, and its improved MRF further introduced an adaptive reweighting mechanism to enhance convergence. The SPA used the vector projection principle to eliminate multicollinearity by greedily selecting the wavelength with the best orthogonality consecutively to construct a low-dimensional, high-information subset of variables [31]. The VISSA facilitated the gradual contraction of the feature space and convergence to the optimal subset by iteratively forming variable subspaces and optimising them according to specific criteria within the framework of model clustering analysis [32]. The ICO algorithm used information-theoretic, variance-based or correlation-based indicators to sort and filter variables [33]. It is often used as a pre-processing step for initial dimensionality reduction. The above techniques are widely used for modelling in infrared spectroscopy and hyperspectral imaging as well as in industries such as agriculture, food and pharmaceuticals. The selection of these methods should account for data dimensionality, noise level, computational resources and model objectives.

### 2.5. Construction and Evaluation of Machine Learning Models

Machine learning algorithms are considered essential for enhancing model performance and robustness. They typically comprise two phases: training and prediction. In the training phase, a portion of pre-existing data (the training dataset) is used by the algorithm to learn the parameters of a model or function, enabling it to make precise predictions or classifications on new data. During the prediction phase, the trained model is applied to new data and the algorithm performs tasks such as prediction and classification. By analysing a specified number of data samples, machine learning algorithms demonstrate exceptional proficiency in separating classes in classification tasks. Generally, such algorithms are classified into supervised, unsupervised and reinforcement learning approaches. Machine learning algorithms are well-suited to a wide range of problems and data types. The selection of the right algorithm can improve the effectiveness of machine learning operations. The selection of the right algorithm can improve the effectiveness of machine learning operations. In addition to the internal test set, three internal validation protocols were conducted: (1) 10-fold stratified cross-validation (CV) on the full dataset, with fold-wise accuracy recorded; (2) bootstrap resampling (1000 iterations) to calculate the 95% confidence interval of the accuracy; (3) Y-randomisation permutation test (100 random label shuffles) to rule out chance-level performance. For each shuffle, the entire optimal preprocessing, feature selection and classification pipeline was reapplied, and the distribution of test accuracies was compared to the original model’s accuracy.

This study thoroughly assessed eight typical methods: backpropagation neural network (BPNN), convolutional neural network (CNN), radial basis function (RBF), random forest (RF), extreme learning machine (ELM), long short-term memory (LSTM), support vector machine (SVM) and particle swarm optimisation–support vector machine (PSO–SVM).

To assess model performance, accuracy, precision, recall and F1 score were calculated for each category. The closer these evaluation metrics were to 100%, the more effective the model was. The performance measures were calculated as follows:

Accuracy = (TP + TN)/(TP + FN + FP + TN),(2)

Precision = TP/(TP + FP),(3)

Recall = TP/(TP + FN),(4)

F1 = 2 × Precision × Recall/(Precision + Recall),(5)

where TP, FP, TN and FN denote true positive, false positive, true negative and false negative, respectively.

### 2.6. Software

MIR spectral data were collected and exported using Spectrum v10.5 software (PerkinElmer, Waltham, MA, USA). Data pre-processing, model creation and machine learning task execution were performed on SIMCA 13.0 and MATLAB R2021b (MathWorks Inc., Natick, MA, USA) Visualisation workflows were conducted using Origin 2025 (OriginLab Corporation, Northampton, MA, USA).

## 3. Results and Discussion

### 3.1. Identification of the Authenticity of AS

#### 3.1.1. MIR Spectral Characteristics of Genuine and Counterfeit AS

**Figure 1:**
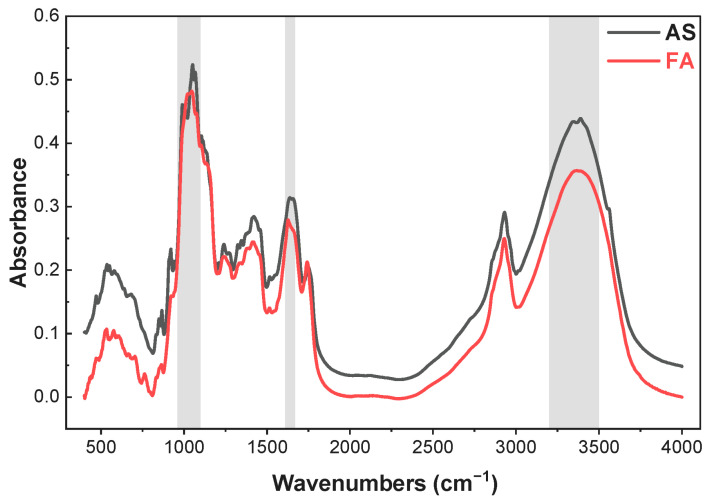
Original mean spectra of *Angelica sinensis* (Oliv.) Diels (**AS**) and fake products (**FA**).

Mid-infrared spectral measurements of authentic and fake AS were obtained over the wavenumber range of 400–4000 cm^−1^. Although the species varied, the average spectral profiles of the samples marked similarity in the distribution of absorption bands, with mostly slight variations (Figure 1). In terms of spectrograms, characteristic peaks observed in the 3300–3400, 1610–1670 and 960–1100 cm^−1^ ranges showed marked differences. The FTIR spectrum of the authentic *Angelica sinensis* (AS) sample is characterized by several broad and prominent absorption bands. The intense, broad band at ~3400 cm^−1^ arises from O–H stretching vibrations of hydroxyl groups, which are abundant in polysaccharides, a major class of components in *Angelica sinensis*, as well as from minor contributions from N–H stretching of amides in proteins [34]. The bands at ~2930 cm^−1^ are assigned to C–H stretching vibrations of aliphatic groups (methyl, methylene) primarily derived from lipids and cuticular waxes [35]. Notably, a well-defined shoulder at ~1740 cm^−1^, attributed to C=O stretching of ester carbonyl groups, is a key feature. This peak is likely associated with esterified phenolic acids, such as ferulic acid, which is a well-known active marker compound in *Angelica sinensis* radix [36,37]. The complex series of strong overlapping peaks in the 1300–1000 cm^−1^ region (notably at ~1105, ~1060, ~1010 cm^−1^) are the signature of C–O and C–O–C stretching vibrations from various polysaccharides, which are a dominant component in *Angelica sinensis* [34,35]. On the basis of expertise and experience, it became challenging to rapidly and effectively determine the authenticity of a large number of samples. Therefore, objective and precise modelling was achieved by applying machine learning techniques.

#### 3.1.2. Construction and Evaluation of Machine Learning Models in Identifying the Authenticity of AS

Based on the Kennard–Stone algorithm, 241 AS samples were assigned to the training group and 73 AS samples were assigned to the prediction group. Furthermore, 27 and 43 fake samples were distributed to the training and prediction groups, respectively. Motivated by the studies of Hu et al. [38] and Wang et al. [39], the initial development of eight machine learning methods, namely BPNN, CNN, RBF, RF, ELM, LSTM, SVM and PSO–SVM, focused on quick determination of the authenticity of AS and the assessment of the effectiveness of various classification models. Meanwhile, considering the issue of class imbalance (314 AS samples and 70 fake samples), F1-score, precision and recall are used to verify the specific impact of the imbalance on the model.

**Figure 2:**
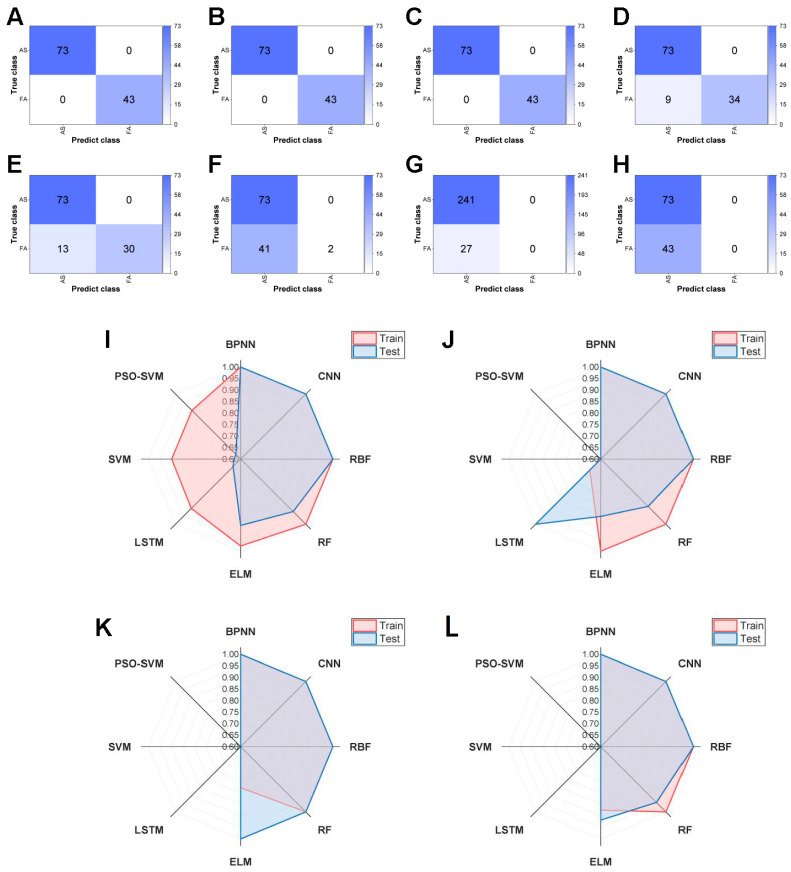
Confusion matrix for the different models in test sets: (**A**) BPNN; (**B**) CNN; (**C**) RBF; (**D**) RF; (**E**) ELM; (**F**) LSTM; (**G**) SVM; (**H**) PSO-SVM. The result of all signal model performance: (**I**) the result of the accuracy value; (**J**) the result of the precision value; (**K**) the result of the recall value; (**L**) the result of the F1 value.

For AS authenticity identification, raw spectral data processed through these machine learning methods yielded remarkable results. Notably, BPNN, CNN and RBF demonstrated the highest accuracy in classifying prediction sets across the training and test sets, with no misclassification in 100% accuracy, precision, recall and F1 score results (Figure 2). The other five machine learning models could not be entirely ruled out; for example, RF and ELM showed excellent potential for authenticity classification, obtaining 92.24% and 88.79% accuracy on the test sets, respectively (Table 1). By contrast, LSTM, SVM and PSO–SVM failed to reach similar accuracy levels.

In conclusion, the most effective modelling classifiers for authenticity identification were BPNN, CNN and RBF. Each demonstrated substantial predictive capability. Although these findings were initially confirmed, they were expected to refine in forthcoming studies.

### 3.2. Identifying Geographical Origins of AS

#### 3.2.1. MIR Spectral Characteristics

**Figure 3:**
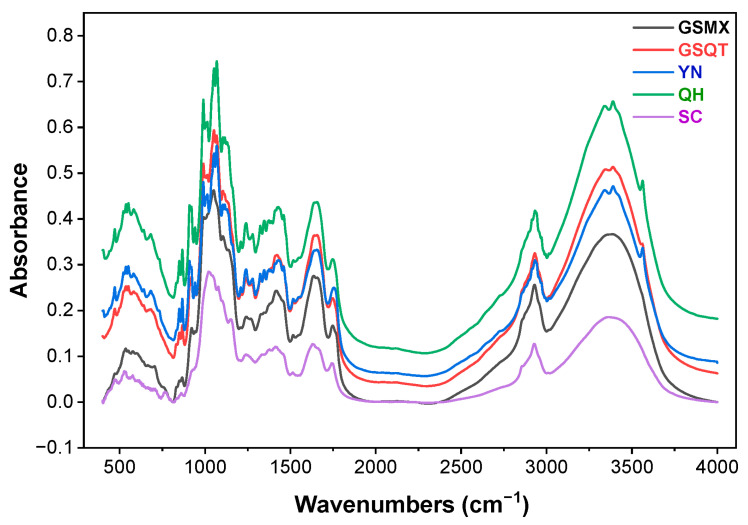
Original mean spectra of *Angelica sinensis* (Oliv.) Diels (AS) from five geographic origins: Min County (**GSMX**), areas in Gansu Province except for Min County (**GSQT**), Qinghai Province (**QH**), Yunnan Province (**YN**), and Sichuan Province (**SC**).

This section primarily discusses the spectral features of MIR with respect to their geographical origins. Despite differences in geographical origin, the waveforms from five locations, namely GSMX, GSQT, YN, QH and SC, exhibited consistent absorption band distributions in the spectrogram (Figure 3), with marked absorption peaks observed at 3600–3300, 2930, 2850, 1745–1733, 1657, 1200 and 950 cm^−1^. The broad band at ~3400 cm^−1^ (O–H stretching) and the aliphatic C–H stretching bands at ~2930 and ~2850 cm^−1^ are present in all spectra, confirming the general chemical composition of polysaccharides, lipids, and proteins; aliphatic C–H stretching bands at ~2928 and ~2854 cm^−1^ (CH_2_ and CH_3_); an ester C=O stretching band at ~1745 cm^−1^ attributed to ferulic acid esters and phthalides (e.g., ligustilide, senkyunolides); amide I (C=O stretching) at ~1655 cm^−1^; amide II (N–H bending) at ~1515 cm^−1^; and strong C–O/C–O–C stretching bands in the 1200–950 cm^−1^ region characteristic of polysaccharides [35,40,41].

#### 3.2.2. Evaluation of Spectral Pre-Processing Methods

**Figure 4:**
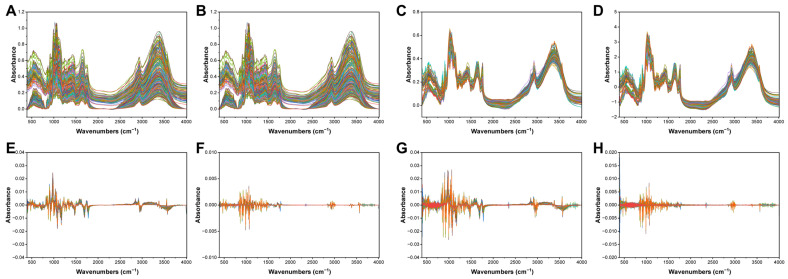
MIR spectra of raw spectrum and AS after preprocessing with seven different methods: (**A**) Raw data; (**B**) Savitzky-Golay smoothing (**SG**); (**C**) multivariate scatter correction (**MSC**); (**D**) Standard normal variate (**SNV**); (**E**) the first derivative (**D1**); (**F**) the second derivative (**D2**); (**G**) Savitzky-Golay second derivative (**SG-FD**); (**H**) Savitzky-Golay second derivative (**SG-SD**).

During spectral acquisition, spectral data remained susceptible to operator variability, instrument noise and environmental factors [42]. To improve the precision of features, pre-processing was crucial for enhancing the effectiveness of subsequent machine learning models, including their resilience and predictive precision [43]. Thus, the unprocessed spectral data were analysed using seven pre-processing techniques: SG smoothing, MSC, SNV transformation, D1, D2, SG-FD and SG-SD. The spectral curves are shown in Figure 4, illustrating variations in spectral profiles.

After implementing SG smoothing as a pre-processing step, the spectrum showed changes in the stability of the baseline and the features of the absorption bands (Figure 4B). The aim of using SG smoothing in pre-processing was to efficiently remove noise while largely preserving the shape of the signal and keeping the peaks intact. Thus, pre-processing with SG smoothing markedly outperformed the raw data in validation tests on the test set (Table 2). However, the results contradicted when the first and second derivatives of the SG-smoothed data were considered (Figure 4G,H). The SG smoothing derivative method computed derivatives by fitting a polynomial to data within a local window, which could further lower background interference and noise. Nevertheless, this approach exhibited notable drawbacks, including the potential introduction of phase delay and the sensitivity of parameter selection. The performance of SG-FD and SG-SD was inferior to that of SG smoothing in specific model but superior in others (Table 2).

Meanwhile, MSC and SNV were selected owing to their suitability for processing solid, granular and irregular samples by effectively lowering scattering interference due to surface irregularities. As demonstrated in certain models, SNV and MSC were prioritised for classification performance, considerably outperforming raw spectra in the test set validation.

D1 and D2 (Figure 4E,F) consistently exhibited the lowest accuracy, with all models demonstrating performance below 60%, thereby highlighting an extremely limited capacity to reduce discriminative spectral features. According to prior studies, D1 effectively removed additive baseline drift; however, they did not address the multiplicative scattering effect. Differentiation markedly reduced the signal-to-noise ratio, amplified high-frequency noise and led to peak-height information loss. Although the D2 process eliminated both additive and linear baselines, it considerably declined the signal-to-noise ratio, improved information distortion and the possibility of the appearance of false peaks.

In conclusion, to enhance the quality of the integrated model and minimise the number of pre-processing techniques employed, D1 and D2 were no longer used. Instead, the remaining five pre-processing methods—SG, MSC, SNV, SG-FD and SG-SD—were retained on the basis of their robustness and predictive accuracy across all machine learning models.

#### 3.2.3. Feature Wavelength Selection

This section describes the application of six characteristic wavenumber selection methods: CARS, ICO, R-Frog, SPA, UVE and VISSA. Table 3 and Table 4 comparatively illustrate the performance of the AS origin identification model under various feature selection strategies, highlighting variations in data characteristics and classification accuracy [44]. Table 3 presents the performance of models using various feature selection strategies, such as raw data, SG smoothing and MSC pre-processing, along with six feature selection algorithms. Conversely, Table 4 presents the performance of models employing different feature selection strategies, such as SNV, SG-FD and SG-SD pre-processing methods, along with six feature selection algorithms. As shown in Table 3 and Table 4, for geographical origin classification of AS, various pre-processing methods combined with feature selection techniques yielded distinct results when applied to both raw and preprocessed spectra using two classifiers.

Models using raw data, combined with various feature selection techniques, declined the prediction accuracy across most models when recognising geographic origins, although to varying extents. A similar trend was observed in models that used spectral pre-processing data combined with feature selection methods. When comparing models constructed via different pre-processing techniques, no considerable improvement in performance was observed after applying pre-processing methods combined with feature selection strategies. This could have happened owing to the elimination of wavenumbers through specific diagnostics contain valuable information.

Among the available model pairs, SG smoothing combined with CARS and BPNN was the most effective. The training and test set accuracies were 99.55% and 98.94%, respectively, with only 147 features selected. Optimising features would yield the best results while reducing computational time and improving efficiency.

**Figure 5:**
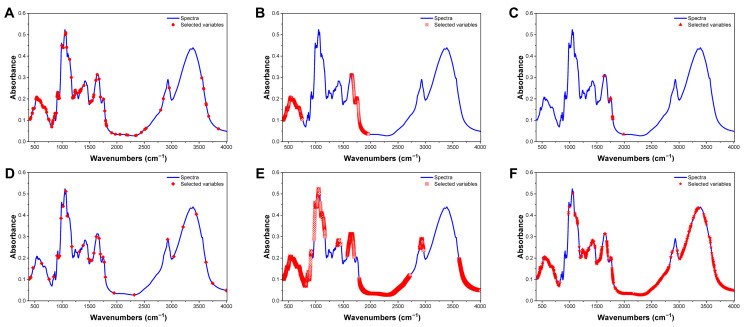
Feature selection results and procedures of CARS (**A**), ICO (**B**), R-Frog (**C**), SPA (**D**), UVE (**E**), and VISSA (**F**) algorithms based on MIR spectra of AS.

In summary, the distribution of selected wavelengths (Figure 5) showed distinct patterns. The CARS, SPA and UVE methods selected more dispersed sets of wavelengths, while the ICO method favoured more concentrated regions. The UVE method emphasised specific wavelength regions, while the VISSA method exhibited a more uniform distribution. Such differences illustrated the diverse feature selection approaches used by each method, ultimately affecting results.

#### 3.2.4. Performance of Identification Models

**Figure 6:**
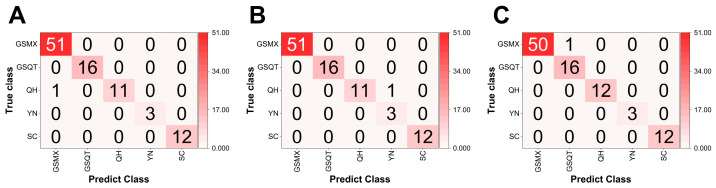
Confusion matrix for the different models in test sets: (**A**) MSC-RBF; (**B**) SNV-RBF; (**C**) SG smoothing-CARS-BPNN.

Among the evaluated models for AS origin identification, the MSC–RBF, SNV–RBF and SG smoothing–CARS–BPNN models were effective spectral pre-processing methods. The MSC–RBF, SNV–RBF and SG smoothing–CARS–BPNN models attained the highest test set accuracies, each with only one misclassification among 44 test samples, corresponding to an accuracy of 98.94% (Figure 6). However, SG smoothing–CARS–BPNN model showed better model performance because precision, recall and F1 score of prediction set were 98.82%, 99.62% and 99.20%, respectively (Table 5). Meanwhile, the 50 repeated random splits yielded a mean test accuracy of 98.2% ± 0.8% (SD), which was not significantly different from the KS-based result (98.94%, *p* > 0.05, paired *t*-test). The 10-fold stratified cross-validation of the optimal model gave an average accuracy of 98.6% ± 0.7%, with fold-wise accuracies ranging from 97.9% to 99.2%. Bootstrap resampling (1000 iterations) produced a 95% confidence interval of [98.2%, 99.5%]. The Y-randomisation permutation test (100 shuffles) resulted in a mean accuracy of only 12.3% (maximum 28.4%), which was far below the original accuracy of 98.94% (*p* < 0.01). These results collectively confirm that the model has learned genuine chemical patterns rather than overfitting to dataset-specific artifacts.

Hence, SG smoothing–CARS–BPNN models was acknowledged as the best techniques for identifying the origin of AS, showcasing their promise as reliable and effective strategies for quick geographic verification of AS.

## 4. Conclusions

To efficiently identify the authenticity and geographical origins of AS, this study systematically evaluated the MIR spectral data of counterfeit AS alongside genuine AS collected from the five primary production regions. Several models were evaluated via diverse pre-processing strategies, wavenumber selection methods and classifiers. By combining raw data on counterfeit and genuine AS with seven classifiers, we identified three optimal modelling approaches—BPNN, CNN and RBF—that achieved a test set accuracy of 100% in classifying AS authenticity. Regarding the determination of AS geographical origins, extensive testing of 72 models using six pretreatment methods, six characteristic wavenumber selection techniques and two parameter optimisation strategies showed that MSC–RBF, SNV–RBF and SG smoothing–CARS–BPNN accurately predicted AS geographical origins, with a prediction set accuracy of 98.94%. Among them, SG smoothing–CARS–BPNN model was the most effective spectral pre-processing method, which precision, recall and F1 score of prediction set were 98.82%, 99.62% and 99.20%, respectively.

Nevertheless, compared with the model outcomes derived from combined pre-processing, the performance of all the integrated models that incorporated both pre-processing and wavelength selection did not considerably enhance. This could be attributed to the integration of pre-processing and wavelength selection, which reduced the advantages of different pre-processing methods, identified more essential wavelengths and lowered the correlation between spectral and actual data. In some respects, techniques such as MSC and SNV reduced the impact of noise on experimental data while maintaining strong model performance. Furthermore, regardless of whether machine learning–based models were used, data pre-processing consistently enhanced model performance. This was particularly effective in the development of origin identification models, where appropriate pre-processing substantially improved the model efficacy.

Despite the rigorous internal validation performed in this study, including repeated random splits, cross-validation, bootstrapping, and Y-randomisation, we acknowledge the absence of a fully independent external validation set as a limitation. Collecting new *Angelica sinensis* samples from the exact same five geographical origins under identical ecological and commercial conditions is challenging due to seasonal constraints and local collection permits, and was therefore not feasible for the current work. Consequently, the reported accuracy of 98.94% for geographical origin discrimination, while robust with respect to the present dataset, should be interpreted as a strong proof-of-concept rather than a directly deployable performance estimate. Future studies should prospectively collect an independent cohort of samples from the same production regions, strictly segregated from the initial dataset during preprocessing, feature selection, and model training, to perform a blind external validation and confirm the real-world generalizability of the proposed strategy.

All binary classification tasks attained over 98% accuracy, demonstrating the efficacy of MIRS combined with machine learning for AS identification. MIR spectroscopy integrated with machine learning not only facilitated rapid and efficient identification of AS medicinal slices but also contributed to maintaining AS quality and enhancing the quality evaluation system. Moreover, it constituted a crucial element of future intelligent production processes for medicinal materials in China.

## Figures and Tables

**Table 1:** The classification results of the machine learning model in the raw data.

Model	Data Set	Accuracy	Precision	Recall	F1
BPNN	Train	1.0000	1.0000	1.0000	1.0000
	Test	1.0000	1.0000	1.0000	1.0000
CNN	Train	1.0000	1.0000	1.0000	1.0000
	Test	1.0000	1.0000	1.0000	1.0000
RBF	Train	1.0000	1.0000	1.0000	1.0000
	Test	1.0000	1.0000	1.0000	1.0000
RF	Train	1.0000	1.0000	1.0000	1.0000
	Test	0.9224	0.8902	1.0000	0.9419
ELM	Train	0.9776	1.0000	0.7778	0.8750
	Test	0.8879	0.8488	1.0000	0.9182
LSTM	Train	0.9030	0.6667	0.0741	0.1333
	Test	0.6466	1.0000	0.0465	0.0889
SVM	Train	0.8993	0.0000	0.0000	0.0000
	Test	0.6293	0.0000	0.0000	0.0000
PSO-SVM	Train	0.8993	0.0000	0.0000	0.0000
	Test	0.6293	0.0000	0.0000	0.0000

**Table 2:** Classification accuracy of AS origin identification based on raw spectra and different preprocessing methods using test set data.

Preprocessing Methods	Classifier							
	BPNN	CNN	RBF	RF	ELM	LSTM	SVM	PSO-SVM
Raw data	0.9255	0.7872	0.9574	0.6915	0.7234	0.8830	0.5532	0.5532
SG	0.9255	0.8298	0.9574	0.6596	0.7979	0.8511	0.5532	0.5532
MSC	0.8404	0.8404	0.9894	0.6596	0.5957	0.8298	0.5532	0.5532
SNV	0.9468	0.8511	0.9894	0.6277	0.6170	0.8298	0.5426	0.5426
D1	0.5426	0.4149	0.4574	0.4362	0.4362	0.3830	0.5532	0.5532
D2	0.5532	0.5103	0.3936	0.5106	0.4468	0.5106	0.5638	0.5638
SG-FD	0.6277	0.8936	0.9255	0.8085	0.6809	0.9362	0.5532	0.5532
SG-SD	0.8936	0.8298	0.8830	0.7978	0.5957	0.9149	0.5426	0.5426

**Table 3:** The performance of AS origin identification models under different feature selection strategies with SG smoothing and MSC preprocessing methods, along with six feature selection algorithms.

Preprocessing Method	Feature Selection	Classifier	Evaluation Indicators		
			Train Set Accuracy	Test Set Accuracy	Number of Features
Raw data	CARS	BPNN	0.9455	0.7553	131
	CARS	RBF	1.0000	0.9362	131
	ICO	BPNN	0.9091	0.7021	679
	ICO	RBF	1.0000	0.9043	679
	R-Frog	BPNN	0.8500	0.7766	10
	R-Frog	RBF	0.8727	0.7021	10
	SPA	BPNN	0.9091	0.7128	39
	SPA	RBF	1.0000	0.9043	39
	UVE	BPNN	0.9591	0.9362	2258
	UVE	RBF	1.0000	0.9468	2258
	VISSA	BPNN	0.8723	0.8723	1243
	VISSA	RBF	1.0000	0.9468	1243
SG smoothing	CARS	BPNN	0.9955	0.9894	147
	CARS	RBF	1.0000	0.9574	147
	ICO	BPNN	0.9091	0.7021	679
	ICO	RBF	1.0000	0.8298	679
	R-Frog	BPNN	0.7909	0.7447	10
	R-Frog	RBF	0.8591	0.7660	10
	SPA	BPNN	0.9545	0.7766	25
	SPA	RBF	0.9955	0.7872	25
	UVE	BPNN	0.9682	0.8830	2283
	UVE	RBF	1.0000	0.9468	2283
	VISSA	BPNN	0.9636	0.7979	1264
	VISSA	RBF	1.0000	0.8830	1264
MSC	CARS	BPNN	0.9773	0.8404	184
	CARS	RBF	1.0000	0.9362	184
	ICO	BPNN	0.9636	0.9574	666
	ICO	RBF	1.0000	0.7766	666
	R-Frog	BPNN	0.8136	0.7766	10
	R-Frog	RBF	0.9000	0.7872	10
	SPA	BPNN	0.9545	0.9043	44
	SPA	RBF	1.0000	0.7979	44
	UVE	BPNN	0.9773	0.9043	2341
	UVE	RBF	1.0000	0.9574	2341
	VISSA	BPNN	0.9727	0.9574	1239
	VISSA	RBF	1.0000	0.9681	1239

**Table 4:** The performance of AS origin identification models under different feature selection strategies with SNV, SG-FD, and SG-SD preprocessing methods, along with six feature selection algorithms.

Preprocessing Method	Feature Selection	Classifier	Evaluation Indicators		
			Train Set Accuracy	Test Set Accuracy	Number of Features
SNV	CARS	BPNN	0.9636	0.8404	184
	CARS	RBF	1.0000	0.9255	184
	ICO	BPNN	0.9636	0.8617	901
	ICO	RBF	1.0000	0.8511	901
	R-Frog	BPNN	0.8364	0.7660	10
	R-Frog	RBF	0.8409	0.7766	10
	SPA	BPNN	0.9455	0.8511	48
	SPA	RBF	1.0000	0.7447	48
	UVE	BPNN	0.9909	0.9043	1080
	UVE	RBF	1.0000	0.8617	1080
	VISSA	BPNN	0.9864	0.9362	1052
	VISSA	RBF	1.0000	0.9149	1052
SG-FD	CARS	RBF	1.0000	0.8085	214
	CARS	LSTM	1.0000	0.9043	214
	ICO	RBF	1.0000	0.8404	1081
	ICO	LSTM	1.0000	0.9255	1081
	R-Frog	RBF	0.9364	0.7872	10
	R-Frog	LSTM	0.8136	0.5851	10
	SPA	RBF	1.0000	0.7872	37
	SPA	LSTM	0.9409	0.7553	37
	UVE	RBF	1.0000	0.9149	1458
	UVE	LSTM	1.0000	0.9468	1458
	VISSA	RBF	1.0000	0.9149	1202
	VISSA	LSTM	1.0000	0.9574	1202
SG-SD	CARS	BPNN	0.9500	0.8723	222
	CARS	LSTM	1.0000	0.9255	222
	ICO	BPNN	0.9364	0.8192	1421
	ICO	LSTM	1.0000	0.9043	1421
	R-Frog	BPNN	0.7955	0.8298	10
	R-Frog	LSTM	0.7545	0.8298	10
	SPA	BPNN	0.6727	0.6064	44
	SPA	LSTM	0.9364	0.6915	44
	UVE	BPNN	0.9182	0.8617	1165
	UVE	LSTM	1.0000	0.9149	1165
	VISSA	BPNN	0.9364	0.7660	1150
	VISSA	LSTM	1.0000	0.9043	1150

**Table 5:** The classification results of RBF and BPNN combined with different pretreatment, characteristic wavelength selection or parameter optimization algorithms.

Preprocessing Method	Feature Selection	Classifier	Training Set	Prediction Set (Macro-Average)			
			Accuracy	Accuracy	Precision	Recall	F1
MSC	-	RBF	1.0000	0.9894	0.9962	0.9833	0.9894
SNV	-	RBF	1.000	0.9894	0.95	0.9833	0.9627
SG smoothing	CARS	BPNN	0.9955	0.9894	0.9882	0.9962	0.9920

## Data Availability

The original contributions presented in the study are included in the article, further inquiries can be directed to the corresponding author.

## Abbreviations

The following abbreviations are used in this manuscript:

AS	*Angelica sinensis* (Oliv.) Diels
MIR	Middle-infrared spectroscopy
GSMX	Min County
GSQT	Various locations in Gansu Province
QH	Qinghai Province
YN	Yunnan Province
SC	Sichuan Province
FA	Fake product
KS	Kennard–Stone
SG	Savitzky–Golay
MSC	Multiplicative scatter correction
SNV	Standard normal variate
D1	First derivative
D2	Second derivative
SG-FD	Savitzky–Golay first derivative
SG-SD	Savitzky–Golay second derivative
CARS	Competitive adaptive reweighted sampling
ICO	Index clear optimise
R-Frog	Random frog
SPA	Successive projection algorithm
UVE	Uninformative variable elimination
VISSA	Uninformative variable elimination and variable iterative space shrinkage algorithm
BPNN	Backpropagation neural network
CNN	Convolutional neural network
RBF	Radial basis function
RF	Random forest
ELM	Extreme learning machine
LSTM	Long short-term memory
SVM	Support vector machine
PSO–SVM	Particle swarm optimisation–support vector machine
